# Lessons learned during COVID-19: Building critical care/ICU capacity for resource limited countries with complex emergencies in the World Health Organization Eastern Mediterranean Region

**DOI:** 10.7189/jogh.11.03088

**Published:** 2021-07-17

**Authors:** Chiori Kodama, Gary Kuniyoshi, Abdinasir Abubakar

**Affiliations:** World Health Organization, Regional Office for the Eastern Mediterranean, Cairo, Egypt

The current COVID-19 pandemic has continued to depict the fragility of public health systems globally by rapidly overwhelming and compromising even robust health structures [1]. Two billion people all over the world are currently living in insubstantial and conflict-affected situations due to complex humanitarian emergencies (CHE) resulting from natural and man-made disasters. The pandemic has affected numerous low-to-middle-income countries (LMICs). COVID-19 response is challenging in these resource-limited (RL) settings combined with CHE, raising concern about the impact of COVID-19 [2,3]. The World Health Organization (WHO) Eastern Mediterranean Region (EMR) comprises 22 countries with a population of nearly 700 million people spread over South East Asia, the Middle East and North Africa. The countries of the region vary greatly in resources, growth indices, and economic strengths [4]. More than 40% of the world’s population facing the impact of complex humanitarian emergencies live in the EMR. The Region is host to a larger number of forcibly displaced people than elsewhere in the world, and more than 70 million people are in need of humanitarian assistance. [5] Besides, fragile health systems have led to suboptimal disease surveillance, preparedness, and response capacities, making the countries of the region immensely vulnerable to the emergence and rapid transmission of novel pathogens, such as COVID-19.

Among those patients that do become symptomatic, most people with COVID-19 develop only mild (40%) or moderate (40%) disease, approximately 15% develop severe disease that requires oxygen support, and 5% have critical disease with complications such as respiratory failure, acute respiratory distress syndrome (ARDS), sepsis and septic shock, thromboembolism, and/or multiorgan failure, including acute kidney injury and cardiac injury [6]. Critical care for COVID-19 is medical care for people who have life-threatening illnesses due to the infection. This includes admission to intensive care units (ICU) and the use of equipment to constantly monitor vital signs as well as giving specialized treatments. The COVID-19 pandemic has required hospitals in numerous countries to expand their surge capacity to meet the needs of patients with severe and critical illness. Public health emergencies have the potential to place enormous strain on health systems in RL countries [7]. As a result, increased mortality has been seen among critically ill patients infected with COVID-19 in such settings [8].

## CRITICAL CARE/ICU CAPACITY IN RESOURCE LIMITED COUNTRIES WITH COMPLEX EMERGENCIES IN EMR

Although building up critical care and ICUs has been one of the most urgently needed investments in RL countries [6,9,10], nonetheless, this has not received proper attention in the EMR due to long and complex conflicts causing fatigue in countries, governments and donors. As a result, the affected countries often seeking quick-fix solutions instead of investing in long-time programs. Resource limited and complex emergency (CE) countries often rely on external support to fill gaps in supplies, equipment and skilled human resources when emergency events occur. Consequently, the required magnitude of surge has substantially exceeded capacity in resource poor and CE settings, necessitating a crisis surge response. [7] On the other hand, the current pace of the pandemic has hindered the deployment of qualified doctors and nurses from abroad [7], mainly due to global movement restrictions and growing needs of human resource within the home country. Therefore, COVID-19 pandemic has engendered an urgent call for response and has necessitated immediate action.

In essence, an ICU needs to be a robust combination of a well-equipped facility and, more importantly, a well-trained multidisciplinary team in RL settings. One of the lessons learned during the COVID-19 response in the region pertains to the use of ICU equipment. For the last one year and six months of the response to COVID-19 pandemic, WHO and partners have procured an enormous amount of biomedical equipment. Unfortunately, we have observed in RL and CE countries that much of this equipment has been left unopened in the warehouse, as local health care workers are unable to properly use them. Biomedical equipment such as ventilators and oxygen delivery devices are essential in ICUs however, they must be operated by trained and experienced medical staff to ensure safety, efficacy, and to prevent adverse events such as ventilator-induced lung injury, hospital acquired pneumonia and in the context of COVID-19, health care worker infections. Additionally, it is important to ensure biomedical equipment is properly maintained in a sustainable manner. We have witnessed that some of this equipment was non-functional and could not be repaired due to the lack of biomedical technicians in the country.

In response to the increasingly recognized demand in human resource development for critical care and ICU management, the WHO has imparted various clinical online and onsite trainings. The programs include an introductory intensive care unit (ICU) training for the fundamentals of critical care management to help build technical and clinical competencies in the management of critically ill patients, and the development of skills in operating life support equipment. These short-term trainings, usually five days for each cohort, have been well received by the countries and indeed played a significant role in the immediate filling of gaps in ICU care by delivering the basics of critical care to non-ICU doctors and nurses. A large number of requests for long-term training programs have been made by the training participants and the local governments upon completion of the initial training. Country ownership of the programs has also been emphasized. Achievements and findings from a short training program provide evidence for the need of RL and CE countries to develop a long-term strategy for critical care capacity building. This includes the development of a specialty training program in critical care with country ownership, multi-organizational support and monitoring.

## A LONG-TERM CRITICAL CARE/ICU TRAINING PROGRAM

Based on the lessons learned during COVID-19, we propose a long-term capacity building program in critical care and ICU management comprising two components:

• Long-term clinical specialty training and mentorship

• Development of operations and management capabilities of ministries of health, host health facilities for ICUs and local institutions

**Figure:**
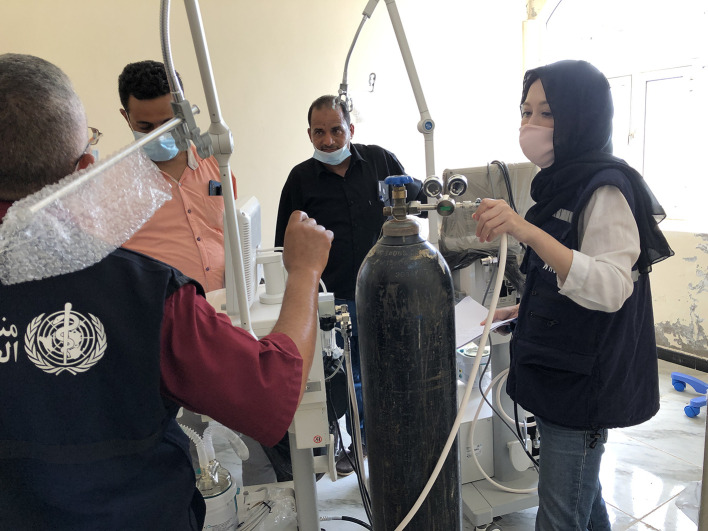
Photo: Providing technical support for the ICU in Yemen. Source: WHO.

in order to strengthen capacity for the provision of quality health care in ICU and critical care in RL and CE countries in the EMR, and to provide technical support to ministries of health. The overall methodology needs to incorporate a continuum critical care approach, eg, bridging pre-hospital and hospital care training programs. The curriculum design of the program should be conducted by working groups: ministries of health, local academic institutions, host hospitals, national health care professionals, WHO and external partners. The delivery of the core curriculum should be through qualified ICU doctors and nurses with dedicated time to train and mentor. Oversight of program implementation, evaluation, monitoring and quality assurance should be done by a local institution with the support of WHO and partners. Throughout the process of designing and implementation, the working group should continuously modify and reinforce the program structure, including advanced degrees and course curricula to best fit the local needs and context. A learning network should be set up to reinforce knowledge and foster academic exchange between national and international academic institutions. Through the implementation of a country support plan in combination with short-term and long-term programs, EMR countries would receive immediate hands-on support for care of the critically ill and injured, develop a country specific training course, country specific ICU SOPs and protocols. The establishment of a core ICU team of local doctors and nurses will serve to provide critical care support to improve health system quality.

## CONCLUSION

It is apparent from the above that urgent action is needed, ensuring the following: integrate explicit ICU health workforce requirements in pandemic response plans, appropriate to its differentiated levels of critical care; ensure proper technical training of all deployed health professionals; ensure sufficient training for ICU Standard Operating Procedures (SOP) and protocols; ensure optimized procurement, training and maintenance of biomedical supplies and equipment for critical and ICU care; ensure safe working conditions in ICU including psychological health and safety of all health workers.

Existing challenges to build comprehensive critical care/ICU capacities for RL countries with complex emergencies in the EMR include political and conflict associated constraints. There is a dire need to undertake a rapid and feasible approach to overcome these barriers. Nonetheless, our ultimate goal for implementation of country support plans in critical care and ICU management across the region is to reduce the case-fatality rate of critically ill and injured patients. Many such patients have been lost during the battle against COVID-19. This is an urgent call for action to initiate a systematic and long-term health system strengthening in critical care and ICU for resource limited and complex emergency countries with full engagement from countries and the global community.
